# Process evaluation of the community-based newborn care program implementation in Geze Gofa district, south Ethiopia: a case study evaluation design

**DOI:** 10.1186/s12884-019-2616-9

**Published:** 2019-12-11

**Authors:** Tsegaye Gebremedhin, Dawit Wolde Daka, Yibeltal Kiflie Alemayehu, Kiddus Yitbarek, Ayal Debie

**Affiliations:** 1 Department of Health Systems and Policy, Institute of Public Health, College of Medicine and Health Sciences, University of Gondar, Po. Box: 196, Gondar, Ethiopia; 2 Department of Health Policy and Management, Faculty of Public Health, Institute of Health, Jimma University, Jimma, Ethiopia

**Keywords:** CBNC, Process, Availability, Compliance, Satisfaction, Evaluation, Ethiopia

## Abstract

**Background:**

The Community-Based Newborn Care (CBNC) program is a comprehensive strategy designed to improve the health of newborns during pregnancy, childbirth, and the postnatal period through health extension workers at community levels, although the implementation has not been evaluated yet. Therefore, this study aimed to evaluate the process of the CBNC program implementation in Geze Gofa district, south Ethiopia.

**Methods:**

A case study evaluation design with a mixed method was employed from May 1 to 31, 2017. A total of 321 mothers who gave birth from September 01, 2016 to February 29, 2017, were interviewed. Similarly, 27 direct observations, six-month document reviews, and 14 key informant interviews were conducted. The quantitative data were entered into Epi-Data version 3.1 and exported to SPSS version 20 for analysis. In the multivariable logistic regression analysis, variables with < 0.05 *p*-values and Adjusted Odds Ratio (AOR) with 95% Confidence Interval (CI) were used to declare factors associated with maternal satisfaction. The qualitative data were transcribed, translated, coded, and analyzed using thematic analysis. The overall process of program implementation was measured based on pre-determined judgmental criteria.

**Results:**

The overall level of the implementation process of the CBNC program was 72.7%, to which maternal satisfaction, availability of resources, and healthcare providers’ compliance with the national guideline contributed 75.0, 81.0, and 68.0%, respectively. Essential drugs and medical equipment, like vitamin K, chlorohexidine ointment, neonatal resuscitation bags, and masks used in the program were out of stock. Very severe diseases were not treated according to the national guidelines, and the identification of neonatal sepsis cases was poor. Trading occupation (AOR: 0.16, 95% CI: 0.03–0.97) and low wealth status (AOR: 3.11, 95% CI: 1.16–8.36) were factors associated with maternal satisfaction.

**Conclusion:**

The process of CBNC program implementation was relatively good, although the compliance of healthcare providers with the national guideline and maternal satisfaction with the services was low. Some essential drugs and medical equipment were out of stock. Merchant and low wealth status affected maternal satisfaction. Therefore, healthcare offices should provide crucial medicines and equipment for better program implementation and improve the wealth status of mothers to enhance maternal satisfaction.

## Background

Proper development and healthy life of babies are fundamental indicators of a country’s socio-economic situation and quality of life [1].

Globally, 2.9 million newborns die, while 2.6 million are stillbirth every year, half of the later occurred during labor [2]. A study conducted in the Morogoro region, Tanzania, showed that 73, 40, 37, and 35% of the cadres of community health workers (CHWs) recalled family planning messages, postpartum care, HIV transmission, and nutrition, respectively. Moreover, 78% of the CHWs were recording information on maternal and child health registers, and 98% were recording data on referral lists at the time of the study [3]. An evaluation conducted in Ghana indicated that 70% of mothers received community-based volunteer visits during their postnatal period, while only 76% of the babies had their respiratory rate, temperature, and weight assessed [4]. A study conducted in Malawi showed that only 36% of women received at least one home visit by health surveillance assistants during their recent pregnancies, whereas 10.9% received post-delivery home visits in the recommended three-days [5].

In Ethiopia, 74% of mothers give birth at home, and 17% of infants receive post-natal care (PNC) in the first 48 h, most of them in urban areas only [6]. Neonatal and postneonatal mortality rates were also 29 and 19 per 1000 live births, respectively [7]. The proportion of deaths in early neonatal and late neonatal periods were 79 and 21%, respectively. Moreover, infant mortality in southern Ethiopia was among the highest in the country. Under-five, child, infant, postneonatal, and neonatal mortalities in the region were 116, 41, 78, 41, and 38 per 1000 live births, respectively [8].

The Ethiopian primary health care units, established at district levels include primary hospitals, local health centers, and five rural health posts. Health Extension Workers (HEWs), who operate at health posts, monitor health programs and disease occurrence, provide health education, essential primary care services and timely referrals to health centers [9–11].

The Health Extension Program (HEP) uses task-shifting and community ownership to provide essential health services at the grassroots using the health development army, a network of woman volunteers organized to promote health, prevent diseases through community participation and empowerment by identifying the salient local bottlenecks which hinder vital maternal, neonatal, and child health service utilization [12, 13].

For improving the survival of newborns in Ethiopia, the Community-Based Newborn Care (CBNC) program has incorporated a platform of HEP, which includes newborn care packages along with the continuum of care from pregnancy to the postnatal period [14]. The community-based newborn care program aims to reduce neonatal mortality through strengthening linkages between health centers and health posts. It also used to improve antenatal, intrapartum, and newborn care through the “four Cs” , prenatal and postnatal contact with the mother and the newborn, case-identification of newborns with signs of possible severe bacterial infection, care or treatment that is appropriate and initiated as early as possible, and completion of a full seven days course of proper antibiotics [15]. The component of the CBNC program was described using the program logic model (Additional file 1).

An evaluation conducted in rural Ethiopia indicated that the implementation of the community-based maternal and newborn health family services by local community-based semi-skilled health workers at village levels was low [16]. Home visit services by HEWs during pregnancy and postpartum periods was lower than what was expected and did not favor the poorest households [17]. Although community-based programs are designed to offer free of charge healthcare services, promote healthy behaviors, screen and refer complicated cases at home or community levels, HEWs are providing the services below the standard in the guideline [18]. Lack of resources and the inaccessibility of healthcare in the rural parts of southern Ethiopia are the main problems of newborn care [19]. In the area, 43.0% of mothers gave birth at health facilities, only 23.0% of the newborns received PNC home visits within seven days, and 7.9% of neonates were identified and diagnosed with sepsis [18]. As a result, healthcare providers compliance with the national guideline in the study area was questionable. However, the implementation status of community-based maternal and newborn interventions has not yet been evaluated. Therefore, this work aimed to evaluate the process of community-based newborn care program implementation in Geze Gofa district, south Ethiopia.

## Methods

### Evaluation design and setting

A case study evaluation design with a concurrent mixed method was used to evaluate the process of community-based newborn care program implementation in Geze Gofa district, Gamo Gofa zone, Southern Nation, Nationalities, and Peoples’ Region (SNNPR), Ethiopia, from May 1 to 31, 2017. Qualitative and quantitative data were collected concurrently.

The district had one urban and 29 rural kebeles (the lowest administrative units) with a total population of 87,731, of whom, 44,041 (50.2%) were female. About 20,441 (23.3%) of the population was in childbearing age (15–49 years), and 3036 women were estimated to be pregnant at the moment. There also were 13,695 under-five children, 2799 infants, and 3036 neonates in the district which owned 29 rural health posts, four health centers, seven private clinics, and 12 drug stores. The study population for the evaluation included mothers who gave birth from September 01, 2016 to February 29, 2017, HEWs’ who are working at health posts in the district for more than a year, head of the district health office, focal persons of the PHCUs, and CBNC service documents.

### Variables and measurements

The formative evaluation approach was used to evaluate the process of the CBNC program implementation. The availability of resources was assessed using a 12 item-indicators to determine whether essential drugs and medical equipment required for the program were supplied or not in the health facilities. The compliance of healthcare providers was also assessed using a 14 item- indicators through measuring the adherence level of the healthcare providers to the national CBNC guideline during the diagnosis or classification and treatment of sick children. Moreover, maternal satisfaction was measured using a 10 item questions, each containing a five-point Likert scale (1 = strongly disagree, 2 = disagree, 3 = neutral, 4 = agree, and 5 = strongly agree) alternatives, and mothers who scored more than 30 points out of the total satisfaction measuring score were considered as satisfied with the CBNC services. The cutoff point for this categorization was calculated using the demarcation threshold formula ($\mathrm{Cutoff}\ \mathrm{point}=\frac{overall\ highest\ score- overall\ lowest\ score}{2}+ total\ lowest\ score$) [20]. The indicators were developed from the national CBNC implementation guideline and other related evaluations [4, 19, 21, 22] using the nominal group technique of stakeholders (Additional file 2).

The process is a set of interrelated activities that lead to the achievement of the goal of the program. Dimensions are essential aspects of the program that the investigator wants to evaluate. Indicators’ weight is the weight given by stakeholders before the evaluation for each selected indicator, and indicator scores were calculated using the formula ($\mathrm{Indicator}\ \mathrm{score}=\frac{\mathrm{observed}\ \mathrm{number}\ \mathrm{X}\ \mathrm{indicator}\ \mathrm{weight}\ }{\mathrm{expected}\ \mathrm{number}}$). The judgment parameter was determined based on the calculated indicator score; the investigator judged 85–100, 75–84.9, 60–74.9, and < 60% as very good, good, fair, and poor, respectively [23].

### Sample size and sampling procedures

The sample for the survey was calculated using the single population proportion formula ($\mathrm{n}=\frac{\mathrm{p}\left(1-\mathrm{p}\right){{\mathrm{Z}}_{\upalpha /2}}^2}{{\mathrm{d}}^2}$) with an assumption that 50% of the mothers were satisfied with CBNC service, 5% margin of error (d), 95% confidence level, and 10% non-response rate. However, the sample was taken from a relatively small population (*N* = 1518). As a result, a finite population proportion correction formula (${\mathrm{n}}_{\mathrm{f}}=\frac{\mathrm{n}}{1+\frac{\mathrm{n}}{\mathrm{N}}}$) was used and gave a final sample of 338. Moreover, all ICCM registration books, integrated maternal and newborn service cards, and administrative records/reports from September 01, 2016 to February 29, 2017, were reviewed. A total of 14 key informant interviews and 27 observations were conducted. Additionally, the medical resources of nine health posts, four health centers, and the district health office were assessed.

Initially, nine health posts were selected using the lottery method [24]. Participants were proportionally assigned to each of the chosen health posts based on their estimated six-month live births. Then, participants were selected by the simple random sampling technique, using their names and house numbers from health post registration books before home visits and interviews were conducted. Three non-participatory observations were carried out to see the interactions among the caregivers of the children and HEWs at each selected health posts. One HEW per health post, all PHCU focal persons, and head of the district health office were selected purposely. Mothers who gave birth in the last six months irrespective of their delivery place, and HEWs who worked at the chosen health posts at least for the preceding of one year were included in the evaluation, but mothers who gave birth in another district, lost their babies, critically ill and unable to respond were excluded.

### Data collection tools and procedures

The standardized structured and the semi-structured versions of the questionnaire were used for the survey and the key informant interviews, respectively. Documents adapted from a variety of evaluations and studies [1, 16, 19, 22, 25–35] were initially developed in English and translated to Amharic, the local language, and back to English to ensure consistency. Four diploma and two BSc degree graduate clinical nurses and two BSc degree graduate public health professionals were recruited for data collection and supervision, respectively, and a day training was given on the basic techniques of data collection and supervision.

Additionally, a pre-test was administered on 17 mothers in Demba Gofa district. The final versions of the tools were modified based on the pre-test findings before the actual data collection. Supervisors checked data accuracy, consistency, and completeness daily. Moreover, the first and the last three observations were dropped per health post to minimize the Hawthorn’s effect. Finally, the qualitative data were analyzed based on the information gained from participants. The manuscript was adhered to the strengthen the reporting of observational studies in epidemiology (STROBE) guidelines.

### Data management and analysis

The quantitative data were cleaned and entered into Epi-Data version 3.1 software and exported to SPSS version 20 for analysis. The binary logistic regression analysis was done, and variables with less than 0.25 *p*-values in the bivariable logistic regression analysis were taken as a candidate for the multivariable logistic regression analysis. In the multivariable logistic regression analysis, a p-value less than 0.05 and AOR with a 95% CI were taken to declare factors associated with maternal satisfaction.

Field notes were properly taken during the key informant interviews, and audio records of key informants were transcribed in the local language and translated to English. Besides, direct observations were carried out to see the interaction between HEWs and clients using a semi-structured questionnaire. The qualitative data were analyzed using thematic analysis through thematizing the availability of resources, compliance of healthcare providers, and the satisfaction of mothers with CBNC services. Finally, each dimension of the process of the CBNC program implementation was judged based on the predetermined judgmental criteria to decide the level of the implementation.

### Ethical consideration

Ethical clearance was obtained from the institutional review board of the College of Health Sciences, Jimma University (Ref. No. IHRPGC/418/2017). Then permission letter was obtained from the district health office and health institutions. After explaining the benefit and risks of the evaluation, informed written consent was obtained from each participant before participation. To ensure confidentiality, names were not used; instead, code numbers were assigned to depict the results, and the questionnaires were kept locked.

## Results

### Availability of resources

There were a total of 138 healthcare workers, of whom 57 (41.3%) were nurses, and 53 (38.4%) HEWs in the district. About 14 nurses and 46 HEWs received training on community-based newborn care. At least one trained HEW was assigned to each health post to provide the community-based newborn care services. Besides, one health care provider was assigned as HEW focal person to supervise each health post.

Key informant interview results indicated that there were low trained health workers and high turnovers of trained HEWs resulting in the interruption of the implementation. All PHCU focal persons and the heads of the health office said that mothers and newborns received no services due to the high turnover of trained HEWs.

*“In the two health posts, HEWs were newly deployed [fresh] and did not receive training, and the other three health posts have one trained HEW [one HEW per health post]. Providing appropriate CBNC services without receiving training is challenging, and the services will be interrupted”* [male clinical nurse, PHCU focal person].

*“I am the only HEW at this health post, and I am not trained on community-based newborn care program. Additionally, there is no alternative way of integrated refresher training on the program. So, this is a barrier that hinders the provision of appropriate services to mothers and newborns”* [mid twenty years old female HEW9].

Essential drugs for the treatment of neonatal sepsis, such as gentamicin 20 mg/2 ml and 125 mg dispersible amoxicillin were available since September 2016 in all health posts. Furthermore, iron with folic acid, tetracycline eye ointment, suppository paracetamol, examination gloves and syringes, iodine solution, and roll bandages were also available. Such drugs and medical supplies have not been out of stock since the provision of services began at each health post. The health posts are provided these medical supplies and drugs every three months continuously by pharmaceutical centers and zonal health departments. Cotton, gauze, and surgical gloves have been available in four health posts since September 2016, but these medical supplies were out of stock at the rest of five health posts. The average stockout days were 80, and the reasons for this were the shortage of resupplies and refills of drugs and medicines from different non-government organizations and the district health office. Moreover, chlorohexidine ointment and vitamin k were stockouts since September 2016 at all health posts.

*“Previously non-government organizations and zonal health department gave us resources, including medicines and supplies which were not sustainable except gentamicin and dispersible amoxicillin. There is also a shortage of budget for the program from the district. So, it is difficult to provide drugs and medical supplies to each health post quarterly”* [male public health nurse, PHCU focal person].

*“Chlorohexidine ointment and vitamin K were not available. Since the beginning of this program, there have been no supply of chlorohexidine and vitamin k; they told us they would give us soon, but it is not available. The program has been going on for more than two years; still, no NGOs and zonal health departments are able to supply”* [mid twenty years old female HEW3].

Resuscitation bags and masks were not available at all health posts. Integrated Community Case Management registration books for young infants (0–2 months), integrated maternal and newborn cards, monthly service delivery reporting formats, services delivery tally sheets, and CBNC key indicator monitoring charts were available in all health posts for recording and reporting of CBNC program. The ICCM Booklet chart used for the treatment of 0–59 months of age was also available.

*“Integrated maternal and newborn service cards, service delivery tally sheets, monthly service delivery report forms, a family guide, ICCM registration books for 0-2 months of age, and other different reporting checklists and booklet charts were available since September 2016, because the zonal health department and district health office were donated annually and in response to our requests, respectively”* [mid twenty years old female HEW4].

However, registration books for home-to-home service delivery and pregnant mothers’ identification and referral form for women health development army team leaders were not available at all health posts (Table 1). The key informant interview results also indicated that these registries were stockouts at health posts.

*“Additionally, the women health development army team leader did not provide the home-to-home service delivery registration books for the CBNC services program and registration books for pregnant mothers identification and referral forms to the health posts as per our request on time. As a result, the home-to-home activities were not recorded and reported properly”* [late twenty years old female HEW6].

**Table 1:** Availability of medicines and supplies of CBNC program in Geze Gofa district health posts, southern Ethiopia, June 2017

Drugs and supplies	Available at the time of observation (*n* = 9)	Available in the last six months (n = 9)
	n (%)	n (%)
Gentamicin 20 mg/2 cc or 10 g/cc	9 (100.0)	9 (100.0)
Dispersible amoxicillin 125 mg	9 (100.0)	9 (100.0)
Fefol (iron with folic acid)	9 (100.0)	9 (100.0)
TTC eye ointment	9 (100.0)	9 (100.0)
Paracetamol suppository	9 (100.0)	9 (100.0)
Examination gloves	9 (100.0)	9 (100.0)
Syringes with needles	9 (100.0)	9 (100.0)
Iodine solution	9 (100.0)	9 (100.0)
Roll bandage	9 (100.0)	9 (100.0)
Neonatal resuscitation bag and mask	0 (0.0)	0 (0.0)
Cotton	4 (45.0)	4 (45.0)
Gauze	4 (45.0)	4 (45.0)
Surgical gloves	4 (45.0)	4 (45.0)
Weighting scale	9 (100.0)	9 (100.0)

The process of the community-based newborn care program in terms of program resource availability was measured to be 81.0%, which was good although it needed improvement based on the judgmental parameter (Table 2).

**Table 2:** Summary of CBNC program resource availability in Geze Gofa district, southern Ethiopia, June 2017

Indicators	E*	O*	W*	S*	A*	JP*
Proportion of HEW trained in CBNC services	53	46	2.7	2.34	86.7	Very good
Proportion of health posts with all HEWs trained in CBNC services	29	22	2.2	2.05	75.8	Good
Number of HC staff trained in CBNC (including CBNC supervision)	85	14	2.0	0.33	16.4	Poor
Proportion of HPs with the existence of continued supply & refill of necessary drug &supplies in the last six months	9	9	2.7	2.7	100.0	Very good
Proportion of HPs with CBNSM medicines (Gentamicin injection and Amoxicillin) in stock during the last six months	9	9	4.0	4.0	100.0	Very good
Proportion of HP with medical supplies (gloves, syringe, cotton, and antiseptics) in stock during the last six months	9	5	2.5	1.4	56.0	Poor
Proportion of HP with functional spring infant scale with sling on the day of assessment	9	9	1.2	1.2	100.0	Very good
Proportion of HP with functional resuscitation bag on the day of assessment	9	0	1.5	0	0	Poor
Proportion of HPs with ICCM registration book for 0–2 months of age on the day of data collection	9	9	1.8	1.8	100.0	Very good
Proportion of HPs with ICCM treatment booklet chart for 0–2 months of age on the day of data collection	9	9	2.0	2.0	100.0	Very good
Proportion of HPs with CBNC implementation guideline on the day of data collection	9	9	1.4	1.4	100.0	Very good
Proportion of HPs with Counseling card /Family Health Card stock in during the last 6 months	9	9	1.0	1.0	100.0	Very good
Overall availability CBNC program resources			25.0	20.22	81.0	Good

^*^E: Expected, O: Observed, W: weight, S: Score ((observed X weight)/Expected), A: Achievement in percentage ((S/W) * 100), JP: Judgement Parameter.

### Compliance with the national guideline

#### Registration and reports

A total of 414 infants less than two months of age were registered on the ICCM registration books. Of the 414 registered infants, about 21, 30, 8, 13, and 4 were diagnosed with severe bacterial infections or very severe diseases, local bacterial infections, pre-term, low birth weight, and feeding problems, respectively. Additionally, 51 of the young infants came to the health posts with fever and fast breathing. Of the 16 young infants classified as very severe diseases and referred to health centers, nine infants received pre-referral treatments. Furthermore, about 23 (76.7%) of the infants were diagnosed with a local bacterial infection and were treated with dispersible amoxicillin 125 mg twice per day for seven consecutive days.

Moreover, a total of 362 mothers visited the health posts and registered for postnatal visits as of September 2016. As a result, 19 mothers were registered in two days of delivery, and about 12 on the third day. Moreover, about 182 mothers were registered between the third and fourth days, 137 women visited the health facility after seven days, while the rest were not recorded. Additionally, 396 (70.5%) live births were identified and registered out of the expected 561 live births from September 01, 2016 to February 29, 2017. Nearly 91% of the weight of the newborns were recorded correctly. Of these, 3.3 and 96.7% of the newborns weighed less than 2.5 kg and 2.5 to 4.5 kg, respectively; 90% of pregnant mothers received first antenatal care visits. An average 10 tasks were provided to sick young infants out of the 16 expected tasks at health posts by the HEWs. As parts of the CBNC services, 74.0, 74.0, 77.8, and 52.0% of the sick young infants’ age, gestational age at birth, weight, and body temperature were measured, respectively. Nearly three-fourths (74.0%) of the participants were asked about the main problems of their sick young infants. Of these, the HEWs asked 74.0% of the caregivers about breastfeeding, 48.0% about convulsion (48.0%), 74.0% about unconsciousness, and 48.0% counted infants’ breathings.

Moreover, the HEWs asked 74.0, 74.0, 52.0, 70.0, and 82.0% of the mothers about vomiting, diarrhea, jaundice, maternal HIV, and immunization status of the sick young infants, respectively. The HEWs correctly classified 78.0% of the sick young infants’ illnesses based on their chief complaints and physical examination findings (Table 3).

**Table 3:** Direct observation result of a sick young infant assessment tasks by HEWs in Geze Gofa district health posts, southern Ethiopia, June 2017

Tasks for a sick young infant	Performed by HEWs (*n* = 27)
	n (%)
Give/show respect for caregivers	27 (100.0)
Sick neonate/young infant’s age asked	20 (74.1)
Assessed for gestational age at birth	20 (74.1
Measure weight	21 (77.8
Measure body temperature	14 (51.9)
Ask the main problem for sick neonate/young infants	20 (74.1)
Ask about young infants is unable to breastfeed	20 (74.1)
Ask for convulsion	13 (48.1)
Ask for unconsciousness	20 (74.1)
Ask for a breathing problem	14 (51.9)
Count breathing per minute	13 (48.1)
Ask for vomiting	20 (74.1)
Ask for diarrhea	19 (70.4)
Ask for jaundice	20 (74.1)
Ask for maternal and child HIV status	20 (74.1)
Ask for immunization status	22 (81.5)

The key informant interview results showed that three-fourths of the health posts received weekly follow-ups and supervisions; 25.0% program-specific supportive supervisions from the zonal health department, while all health posts were received quarterly integrated supportive supervision from the district health office.

*“I received integrated non-program specific supervision from the health center and woreda health office supervisors and quarterly CBNC program-specific supportive supervision form the zone, and the last supervision was on December 17, 2016. I also had woreda health office supervision every three months, and the last integrated supportive supervision was in January 2017. Earlier I had weekly supervisions from the health center which is interrupted at the moment”* [late twenty years old female HEW6].

The survey result showed that all of the respondents knew the HEWs, and the majority of the mothers received advice during their recent pregnancies and in the postpartum period. About 277 (94.0%) mothers were members of women’s health development team, and 56.0% attended meetings during their recent pregnancies. The mean age of the pregnancy during their last ANC visit was 4.6 months (± 1.2 SD). Half (50.4%) of the mothers received advice during the second and 2.3% during the third trimester. More than three fourths (84.4%) of the mothers attended their antenatal care at health posts; 274 mothers took iron for an average of 68 (IQR: 15–180) days during their recent pregnancies. Furthermore, 79.1 and 68.8% of the mothers knew vaginal bleeding, and severe headaches were danger signs during pregnancy, respectively. Almost half (53.0%) of the mothers delivered at health centers and 2.8% at the health posts. Of those health post deliveries, 9.0% were assisted by HEWs.

About 3.1% of the cord of the newborns were applied to things, such as dungs, powder, or oil immediately after birth by the caretakers. For the prevention of cord infections, 62.3% of caregivers applied particular medicines when available.

More than 90% of mothers started breastfeeding within the first hour. Three-fourths of the mothers received information about breastfeeding from HEWs. From the total respondents, 70.4% of mothers were given information about CBNC by HEW at health posts.

About 88.8, 75.4, and 70.1% of mothers knew fever, poor sucking or inability to suck and fast breathing were the danger signs of newborns, respectively. Within the first two months of delivery, about 60.1% of the newborn had their health status checked by HEWs, and 18.1% were verified only once. Besides, nearly 38% of the newborns were checked within 48 h and 21.2% after the seventh day.

Only 15.9% of the newborns experienced at least one health problem in the first two months of age. The mean age of newborns who experienced health problems was 40 (±12 SD) days. Two-thirds of mothers consulted HEWs and visited health posts five times on average to get services for their newborns. Two hundred sixty-two newborns were weighed within seven days, and 88.9% in the first 24 h; 81.3% were considered at the health centers. Additionally, 54.5% of the mothers said that the availability of drugs in the health posts was convenient. Overall, 68.0% of the HEWs complied with the national guidelines during the provision of CBNC services (Table 4).

**Table 4:** Summary of performance indicators of compliance to national CBNC guidelines in Geze Gofa district, southern Ethiopia, June 2017

Indicators	E*	O*	W*	S*	A*	JP*
The proportion of pregnant women who received at least one ANC by HEWs at HP in the last six months	561	506	3.1	2.8	90.0	Very good
The proportion of live births identified from HH level in the catchment area in the last six months	561	396	3.1	2.2	70.5	Fair
The proportion of newborns who received PNC visits by HEWs within 48 h in the last six months	561	193	3.1	1.1	34.5	Poor
The proportion of sick young infants who assessed for more than 12 tasks from16 tasks during the observation	27	13	3.1	1.5	48.0	Poor
Proportion of sick young infants who are asked for main problem/chief compliant	27	20	3.0	2.2	74.0	Fair
The proportion of neonatal sepsis cases who are treated in a given catchment area in the last six months	44	44	4.7	4.7	100.0	Very good
The proportion of neonatal sepsis cases who received treatment in a given catchment area by HEWs in the last six months	51	30	4.7	2.8	59.0	Poor
The proportion of very severe diseases (VSD) treated with an initial dose with appropriate antibiotics in the last six months	21	14	4.7	3.1	66.0	Fair
Proportion of VSD referred by HEWs in the last six months	21	16	3.2	2.4	76.0	Good
The proportion of VSD receiving 7 consecutive days of gentamycin in the last six months	21	5	4.7	1.2	24.0	Poor
The proportion of treated neonatal sepsis cases whose treatment outcome has improved in the last six months	28	23	4.7	3.9	86.0	Good
The proportion of NS cases referred by HEW in the last six months	51	16	3.7	1.2	31.0	Poor
The proportion of neonatal sepsis cases correctly classified in the last six months	51	40	4.6	3.6	78.0	Good
The proportion of neonatal sepsis cases correctly treated in the last six months	40	32	4.6	3.7	80.0	Good
Overall compliance of HEWs			55	37.4	68.0	Fair

^*^E: Expected, O: Observed, W: weight, S: Score ((observed X weight)/Expected), A: Achievement in percentage ((S/W) * 100), JP: Judgement Parameter.

### Maternal satisfaction with CBNC services

#### Socio-demographic and economic characteristics of mothers

A total of 321 women responded to the interviewer-administered questionnaire with a response rate of 95%. The age of mothers in the study ranged from 18 to 42 years and their mean age was 27.6 (SD ±5) years. About 75.7% of mothers were married; 46.7% were Protestant Christian; 43.9 and 71.7% were elementary school leavers and housewives, respectively, and 30% were in the middle quantile of wealth status (Table 5).

**Table 5:** Socio-demographic and economic characteristics of mothers in Geze Gofa district, southern Ethiopia, June 2017 (*n* = 321)

Variables	Frequency (n)	Percent (%)
Age in years		
< 24	95	29.6
24–35	211	65.7
> 35	15	4.7
Marital status		
Single	16	5.0
Married	243	75.7
Widowed	27	8.4
Divorced	35	10.9
Religious status		
Protestant	150	46.7
Orthodox	112	34.9
Muslim	28	8.7
Catholic	31	9.7
Educational status		
Unable to read and write	99	30.8
Able to read and write	21	6.6
Elementary school (up to grade 8)	141	43.9
High school	42	13.1
College and above	18	5.6
Occupational status		
Gov’t employee	13	4.0
Merchant	25	7.8
Daily labor	21	6.5
Farmer	32	10.0
Housewife	230	71.7
Ethnicity		
Gofa	216	67.3
Gamo	55	17.1
Wolayita	26	8.1
Others*	24	7.5
Parity		
Primipara	53	16.5
Multipara	268	83.5
Wealth quantiles		
Lowest	55	17.1
Second	54	16.8
Middle	97	30.2
Fourth	47	14.7
Highest	68	21.2

*Amhara, Guraghe, Kembata.

#### Maternal satisfaction

The finding showed that more than half (52.3%) of the mothers were satisfied with the CBNC counseling services; about 181 (56.4%) were satisfied with the appropriateness of the visiting time for CBNC services. The level of maternal satisfaction with the appropriateness of the consultation time was 151 (47.0%). Furthermore, 137 (42.7%) mothers were satisfied with the knowledge/competency of HEWs; 31.0% with the availability of drugs at health posts; nearly half (45.2%) were satisfied with the courtesy of HEWs. The overall level of maternal satisfaction with the community-based newborn care services was 75.0% (Table 6).

**Table 6:** Summary of performance of satisfaction indicators for CBNC program in Geze Gofa district, southern Ethiopia, June 2017

Indicators	E*	O*	W*	S*	A*	JP*
The proportion of mothers who satisfied with the counseling services they received.	321	225	2.0	1.4	70.1	Fair
The proportion of mothers who perceive visiting time to receive CBNC service is appropriate/good	321	265	2.0	1.7	82.6	Good
The proportion of mothers who perceive consultation time about CBNC service is appropriate/good	321	271	1.7	1.4	84.4	Good
The proportion of mothers who perceive CBNC service providers (HEWs) are competent	321	285	2.7	2.4	88.8	Very good
Proportion of mothers who perceive who receive convenient appointment date/time	321	260	2.0	1.6	81.0	Good
The proportion of mothers who perceive the working hour of health post are convenient	321	249	2.0	1.6	77.6	Good
The proportion of mothers who perceive the available drugs are enough at the health post.	321	192	3.3	2.0	59.8	Poor
The proportion of mothers who perceive the approach of HEWs is good.	321	217	1.7	1.1	67.6	Fair
The proportion of mothers who perceive the waiting area of health post are clean	321	225	1.7	1.2	70.1	Fair
The proportion of mothers who perceive the waiting area of health post are appropriate	321	229	1.0	0.7	71.3	Fair
Overall compliance of HEWs			20.0	15.0	75.0	Good

^*^E: Expected, O: Observed, W: weight, S: Score ((observed X weight)/Expected), A: Achievement in percentage ((S/W) * 100), JP: Judgement Parameter.

#### Factors associated with maternal satisfaction

In the multivariable logistic regression analysis, occupational status and wealth quintiles were significantly associated with maternal satisfaction with CBNC services. Accordingly, merchant mothers were 84.5% less likely to be satisfied than government-employed mothers (AOR: 0.15, 95% CI: 0.02–0.97). Moreover, mothers in the second quantiles of wealth status were three times more satisfied compared to those in the lowest quantiles of wealth status (AOR: 3.11, 95% CI: 1.16–8.36) (Table 7). Finally, the overall process of the community based newborn care program implementation was 72.7%, as measured by the availability of medical equipment and drugs, healthcare providers’ compliance with the national guideline and maternal satisfaction.

**Table 7:** Bivariable and multivariable logistic regression analysis for the satisfaction of mothers on CBNC services in Geze Gofa district, southern Ethiopia, June 2017 (n = 321)

Variables	Satisfaction category		COR	AOR (95% CI)
	Satisfied	Dissatisfied		
	n (%)	n (%)		
Age in years				
< 24	72 (22.4)	23 (7.1)	1	1
24–35	145 (45.2)	66 (20.6)	0.702	0.72 (0.39–1.30)
> 35	11 (3.4)	4 (1.3)	0.878	1.35 (0.35–5.16)
Marital status				
Single	9 (2.8)	7 (2.2)	1	1
Married	171 (53.3)	72 (22.4)	1.847	2.15 (0.68–6.82)
Widowed	22 (6.9)	5 (1.6)	3.422	3.29 (0.71–15.27)
Divorced	26 (8.0)	9 (2.8)	2.247	2.26 (0.56–9.12)
Occupational status				
Gov’t employee	11 (3.4)	2 (0.6)	1	1
Merchant	15 (4.7)	10 (3.1)	0.273	0.15 (0.03–0.97) *
Daily labor	18 (5.6)	3 (1.0)	1.091	0.75 (0.09–6.11)
Farmer	25 (7.8)	7 (2.2)	0.649	0.40 (0.06–2.65)
House wife	159 (49.6)	71 (22)	0.407	0.21 (0.04–1.13)
Wealth quintile				
Lowest	37 (11.5)	18 (5.6)	1	1
Second	46 (14.3)	8 (2.5)	2.797	3.11 (1.16–8.36) *
Middle	68 (21.1)	29 (9.0)	1.141	1.03 (0.47–2.24)
Fourth	30 (9.3)	17 (5.3)	0.859	0.79 (0.32–1.98)
Highest	47 (14.6)	21 (6.5)	1.089	1.07 (0.47–2.47)
Drug availability				
Yes	118 (36.8)	57 (17.8)	1	1
No	110 (34.3)	36 (11.1)	1.476	1.48 (0.87–2.54)

*statistically significant at p-value < 0.05.

## Discussion

In this evaluation, the formative approach with driven indicators was used to measure the process of CBNC program implementation. The overall process of CBNC program implementation in the district was 72.7%. The structure component was 81.0% as measured by the availability of resources. The compliance of HEWs with the national guideline and the satisfaction of mothers was 68.0 and 75.0%, respectively. The process of the program implementation needed some improvements according to the judgment parameter.

The findings of our evaluation showed that the program resources, like gentamicin, amoxicillin, and iron were 100.0% available in the district health posts to the minimum standards according to the CBNC implementation national guideline whereas, cotton, surgical gloves, and gauze were available in 45.0% of the health posts. On the contrary, drugs and medical supplies, like chlorohexidine ointment, vitamin k, and resuscitation bag were stockouts for more than six months. This stockout was due to the absence of resupply from the pharmaceutical center and other NGOs since the beginning of the program implementation in the district, and no budget has been explicitly allocated for the program.

Moreover, there were low trained HEWs due to the high turnover rate and the absence chances of upgrading in the field of study. The other possible reason might be that there was no alternative mechanisms of training for newly assigned HEW’s and other healthcare providers at the health center and the health office. Moreover, CBNC training was not included in the integrated refreshment training of the HEW program. The finding is in line with that of a study conducted in Asia and Africa at clinical-care level and shows that the limited number of human resources was the predominant challenge for maternal, newborn, and child health service delivery [36].

All health posts had ICCM registration books, ICCM chart booklets, integrated maternal and newborn service cards, and CBNC key indicator monitoring charts. According to the Ethiopian national CBNC guideline, the minimum required program resource for health posts to provide CBNC services includes human resources (trained), logistics and supplies, and supportive systems [19].

Our evaluation findings are higher than that of a study done in Ethiopia indicated that 90.0% of health posts had ICCM registration books and 65% had ICCM chart booklets. Moreover, clean gloves, syringes with needles, gentamycin and tetracycline eye ointment were available in 64.0, 80.0, 64.0, and 80.0% of the health posts, respectively. Approximately 80.0% of the health posts had family folders (a family-centered information collection tool for integrated health service delivery by HEWs) and registration books for pregnant women and outcomes [29, 37].

Our evaluation findings showed that the number of trained HEWs was relatively low. The inadequacy of trained health care providers had the implication that service provisions were below the required amount for target beneficiaries. This finding is comparable with that of an evaluation conducted in Zambia and showed that deficiencies in interventions reported included poor ongoing support and inadequate supplies needed for safe motherhood action groups to facilitate their work [27].

The findings showed that 68.0% of the CBNC services were implemented according to the national standards. In the process of service delivery, there was an outstanding achievement of provisions of first ANC and neonatal sepsis treatment. But delivery attendance by HEWs in cases of emergency and the identifications of live births at household levels in the catchment area were poor. This might be explained by the inadequacy of the home-to-home visits by HEWs and weekly supportive supervisions from respective catchment health centers.

Our finding showed that three-fourths of the health posts received weekly follow ups and supervisions from the health centers irregularly. But all health posts received quarterly integrated supportive supervisions from the district health office. Moreover, a quarter of the health posts received program-specific supportive supervisions from the zonal health department. These findings were comparable with the results of the baseline evaluation of CBNC in Ethiopia and revealed that 82% of the HEWs received quarterly supportive supervisions. Of these, 86% were visited by their supervisors from the health centers and a half from woreda health offices. On average, the HEWs received five supervisory visits within three months. Less than half of the supervisory visits covered discussions on newborn care services [31, 38]. The result was lower than that of a study conducted in Morogoro region, Tanzania, and showed that facility-based providers were supported the CHWs more than half from the national standards in three months [3]. But higher than that of an evaluation conducted in Zambia and revealed that most safe motherhood action groups expressed concern over lack of supervision and refresher courses from the health facility and district health staff [27].

Our evaluation showed that 94% of the women participated in the women’s health development teams during their recent pregnancies. The result is in line with the national guideline [19]. In this evaluation, 51.9% of the sick young infants had their body temperature measured and managed for hypothermia. The result was higher than that of a study conducted in Ethiopia (COMBINE aggregated data) and showed that 43% of the newborns received management of hypothermia [29].

Moreover, the findings of our evaluation showed that 65.0% of the newborns did not receive the first postnatal HEW visits within 48 h. The result might be due to the high coverage of home delivery, inadequacy of HEWs and infrequent home-to-home visits. This result was higher than a finding in Malawi which showed that 36.0% of the women received at least one pregnancy home visit by HSA, while only 10.9% of newborns received a post-delivery home visit within the recommended 3-days [39]. But the analysis of routine COMBINE aggregated data in Ethiopia showed that 62.0% of newborns were visited by HEWs within 48 h of birth [29]. The variation might be due to differences in study setups and sample sizes. The finding was lower than the national guideline of CBNC recommendation. A mother who gives birth, irrespective of the birth place, should get the home-to-home visit within 48 h by HEWs and get registered on the ICCM registration book [19].

Our evaluation showed that 91.6% of the mothers were aware of the health development team, and 58.6% attended meetings in the community during their recent pregnancies. The finding was lower than the national standards, which recommends that all mothers should participate in the women’s health development team meetings during their pregnancies. This might be explained by the presence of weak 1 to 5 women networks in the community. The finding was higher than an evaluation result in Malawi which revealed that 34% of the women were aware of the core groups in their community, although the groups did not make regular visits to pregnant women as only 9.6% received home visits from a core group [5].

In this evaluation, 45.5% of the sick neonates with very server diseases were referred with an initial dose of gentamicin and dispersible amoxicillin, 31.8% were referred without an initial treatment, and 22.7% were treated at health posts with gentamicin 20 mg/2 ml daily and 125 mg dispersible amoxicillin twice per day for seven consecutive days. This finding was incongruent with the national standards. Infants less than two months of age with very severe bacterial infections should be referred to health centers or hospitals with a pre-referral gentamicin treatment, and if the referral is impossible, they should be treated with gentamicin 20 mg/2 ml/kg daily and dispersible amoxicillin 125 mg twice per day for seven consecutive days at the health post [40]. This low adherence to the national guideline might be due to weak follow-ups and supervisions of HEWs from the catchment health center. This result was in line with a study in Bangladesh which showed that 34, 43, 5, and 18% of the sick neonates were referred successfully, treated by CHWs in case of unsuccessful referrals, received care from another source, and received no care, respectively [34].

Our evaluation showed that 68.0% of the CBNC services were implemented in line with the national standards. This finding was higher than that of an evaluation conducted in Pakistan and revealed that many of the newborn care services were not consistent with the recommended practices [41].

In this evaluation, the satisfaction of mothers with the availability of drugs at health post, courtesy of HEWs, counseling services by HEWs, cleanness, and appropriateness of health post waiting area was 59.8, 67.6, 70.1, 70.1, and 71.3%, respectively. Moreover, the findings showed that merchants were more likely to be associated with dissatisfaction than government employees, while those in the second wealth quintile were more likely to be satisfied than those in the lowest. The low satisfaction level of mothers with low wealth status showed that home-to-home service provision for the poorest was still low and incongruent with the guideline. Our finding was in line with that of a study done on community perception and health-seeking behaviors in southern Ethiopia and showed that the coverage of home visits by HEWs during pregnancy and postpartum period was lower than the expected and generally did not significantly favor the poorest households [1].

Moreover, inconvenient time of services to merchant mothers might have made the services unsatisfactory. This might also be explained by the fact that government employees had more information about the services than others. This finding is in line with that of an evaluation in Ghana and showed that CHW visits were welcome and acceptable to families who were pleased with the assessment visits which were considered as reassuring to the state of health of their newborns as caregivers were convinced that CHWs were knowledgeable [4]. Another study in Uganda revealed that mothers accepted and welcomed CBNC intervention to improved their own care and that of their newborn babies through home visits to households by health workers during pregnancy and in the first week after delivery [33, 42]. Moreover, the finding was in line with the result of an evaluation conducted in rural Nepal and showed that interpersonal peer communication through community-level volunteers promoted household-level behaviors was acceptable to clients and health workers [22]. The satisfaction of mothers with the overall process of CBNC services was 75.0%, which is fair and needed improvement.

### Strengths and limitations of the evaluation

Our work used three dimensions to evaluate the process of CBNC implementation which makes it more valid than measuring the process by a single dimension. Besides, using both qualitative and quantitative methods (triangulation) also helped us get accurate and detailed results.

The possible limitation of this evaluation was the hawthorn effect, which might have occurred during the direct observation of the interactions between clients and HEWs. To minimize this, we dropped the first and last three observations. Recall bias of caregivers about their history of pregnancy before six months, especially for their first ANC visit was another limitation, but compared to other studies the duration was not so long as to create recall problems. The improper registration of all of the services delivered was an additional limitation of the evaluation for the assessment of health care providers’ compliance.

## Conclusion

The primary health care units in Geze Gofa district had the least required resources to provide CBNC services to mothers and newborns at home and the health post level. The numbers of trained HEWs and supervisors were not adequate to implement the program. Essential drugs and medical supplies, like gentamicin, dispersible amoxicillin, iron with folic acid, weight scales, examination gloves, vitamin A for mothers who have delivered and syringes were stock-in. But cotton, surgical gloves and gauze, vitamin k, chlorhexidine ointment, resuscitation bags, and sterilized delivery kits were stockouts. The compliance of the HEWs to the national guideline was poor.

Moreover, the satisfaction of mothers with the process of CBNC services provided by HEWs was relatively good. Trading occupation and low wealth status were factors affecting maternal satisfaction. The availability of equipment and supplies to effectively utilize education/skills should be part of all training programs. Therefore, healthcare offices should provide essential drugs and equipment for better program implementation and improve the wealth status of the mothers to enhance maternal satisfaction with the program.

## Supplementary information

**Additional file 1.** The Logic model of community-based newborn care program in Geze Gofa district, southern Ethiopia, June 2017.

**Additional file 2.** Stakeholders identification and analysis matrix for the process evaluation of community-based newborn care program implementation in Geze Gofa district, southern Ethiopia, June 2017.

## Data Availability

Data will be available upon reasonable request from the corresponding author.

## Abbreviations

ANC: Antenatal Care
AOR: Adjusted Odds Ratio
CBNC: Community-Based Newborn Care
CI: Confidence Interval
COMBINE: Community-Based Interventions for Newborns in Ethiopia
HCPs: Health Care Providers
HEP: Health Extension Program
HEWs: Health Extension Workers
HIV: Human Immunodeficiency Virus
ICCM: Integrated Community Case Management
NGOs: Non-governmental Organizations
NMR: Neonatal Mortality Rate
PHCU: Primary Health Care Unit
PNC: Postnatal Care
PNMR: Post Neonatal Mortality Rate
WHO: World Health Organizations
