# Supplementary material for: Process evaluation of the community-based newborn care program implementation in Geze Gofa district, south Ethiopia: a case study evaluation design
Source: BMC Pregnancy Childbirth. 2019 Dec 11;19:492. doi: 10.1186/s12884-019-2616-9 (PMC6907260; doi:10.1186/s12884-019-2616-9)
Supplement: Supplementary file 1 — Additional file 1. The Logic model of community-based newborn care program in Geze Gofa district, southern Ethiopia, June 2017. [file 12884_2019_2616_MOESM1_ESM.doc]

**Input**

**Activities**

**Output**

**Outcome**

**Impact**

Early Pregnancy identification

# pregnant mothers identified

Human resources

Financial resources

Infrastructures

Medical equipment

Drugs and medical supplies

Guidelines, manuals, recording and reporting formats

IEC/BCC materials

Focused ANC

# pregnant mothers receiving ANC services

Notification of Labor and delivery at community/ household level

# of labor &delivery identified at HH level

**Increase care givers satisfaction**

Assist clean and safe delivery at HP in case of emergency and postnatal care

**Increased utilization of CBNC service**

**Reduction of neonatal morbidity and mortality**

# of delivery assisted & referred by HEWs

# of mothers receiving PNC & referred

Identify sick newborn at community level

# of sick newborns identified by HEWs

# of neonatal sepsis cases treated and care givers counseled

manage neonatal sepsis at community level and counsel caregivers

**Improved caregivers’ awareness**

Community mobilization and health education

# of health education session conducted

**Improved quality of CBNC services**

# of Community conversation conducted

**Improved quality of data**

Report time lines & completeness

Training to HEWs

Training to supervisors and PHCU focal persons

Recording and reporting

# of trained HEWs

# of trained supervisors and PHCU focal persons

**Improved knowledge and practice of HCPs**

Figure 1: Logic model of CBNC program in Geze Gofa district, Southern Ethiopia June 2017
