# Supplementary material for: Process evaluation of the community-based newborn care program implementation in Geze Gofa district, south Ethiopia: a case study evaluation design
Source: BMC Pregnancy Childbirth. 2019 Dec 11;19:492. doi: 10.1186/s12884-019-2616-9 (PMC6907260; doi:10.1186/s12884-019-2616-9)
Supplement: Supplementary file 2 — Additional file 2. Stakeholders identification and analysis matrix for the process evaluation of community-based newborn care program implementation in Geze Gofa district, southern Ethiopia, June 2017. [file 12884_2019_2616_MOESM2_ESM.doc]

Additional file 2: Stakeholders identification and analysis matrix for evaluation of community-based newborn care program implementation in Geze Gofa district, Southern Ethiopia June 2017.

| Stakeholders’ | Role in the program | Interest/perspective on evaluation | Role in evaluation | Ways of communication | Level of importance* |
| --- | --- | --- | --- | --- | --- |
| Geze Gofa district health office | Planning  Implementation  Coordinate and facilitate  Supportive supervision and monitoring  Resource allocation | To Identify skill gap of implementers  To know the implementation status of CBNC services  To learn from experience | Source of information  Formulating evaluation questions and deciding on the focus of evaluation  Facilitation evaluation process  Identify indicators  Assign weight for indicators and set judgment parameters | Formal letter  Face to face  Phone | High |
| Geze Gofa district Health centers | Supportive supervision and review meeting  A timely and complete report  Strength linkage of PHCU | To identify the skill gap and for improvement of knowledge on CBNC program  To identify the weakness and strength of program implementation | Source of information  Formulating evaluation questions and deciding on the focus of evaluation  Facilitation of the evaluation process  Identify indicators  Assign weight for indicators and set judgment parameters | Phone  Face to face | High |
| HEWs | Service delivery  The timely and complete report  Strength linkage of PHCU | To identify the skill gap and for improvement of knowledge on CBNC program  To identify the weakness and strength of program implementation | Source of information  Facilitation of the evaluation process  Identify indicators  Assign weight for indicators and set judgment parameters | Phone  Face to face | High |
| Direct beneficiaries (mothers and young infants) | Service users | For program improvement | Source of information  Formulating evaluation questions | Face to face | High |
| Woreda administration | Budgeting  Facilitation of services  Community mobilization | Identification of services gap | Facilitation evaluation process (transportation) | Phone  Face to face | Low |
| Kebele council | Community mobilization  Create a supportive environment through strengthening HDA and 1 to 5 networks | To know the level of implementation | Facilitation of evaluation (assign volunteers for helping data collector by showing selected mothers’ home) | Face to face  Phone | Low |
| HAD/ 1 to 5 network leaders | Awareness creation  Community mobilization | To know the level of implementation | Source of information  (information about how they identify& refer pregnant mother) | Face to face | Medium |

*Note: - Stakeholders level of importance was rated based on: -

Low– the stakeholder can have done little to adversely affect the outcome of the evaluation.

Medium – the evaluation was achieved its objectives against this stakeholder’s opposition, but it was not easy

High – the person or group significantly changed the evaluation outcome
